# Place Recognition through Multiple LiDAR Scans Based on the Hidden Markov Model

**DOI:** 10.3390/s24113611

**Published:** 2024-06-03

**Authors:** Linqiu Gui, Chunnian Zeng, Jie Luo, Xu Yang

**Affiliations:** 1 School of Information Engineering, Wuhan University of Technology, Wuhan 430070, China; guilinqiu@whut.edu.cn; 2 School of Automation, Wuhan University of Technology, Wuhan 430070, China; zengchn@whut.edu.cn (C.Z.); yx_auto@whut.edu.cn (X.Y.)

**Keywords:** place recognition, global descriptor, hidden Markov model

## Abstract

Autonomous driving systems for unmanned ground vehicles (UGV) operating in enclosed environments strongly rely on LiDAR localization with a prior map. Precise initial pose estimation is critical during system startup or when tracking is lost, ensuring safe UGV operation. Existing LiDAR-based place recognition methods often suffer from reduced accuracy due to only matching descriptors from individual LiDAR keyframes. This paper proposes a multi-frame descriptor-matching approach based on the hidden Markov model (HMM) to address this issue. This method enhances the place recognition accuracy and robustness by leveraging information from multiple frames. Experimental results from the KITTI dataset demonstrate that the proposed method significantly enhances the place recognition performance compared with the scan context-based single-frame descriptor-matching approach, with an average performance improvement of 5.8% and with a maximum improvement of 15.3%.

## 1. Introduction

One of the core technologies for the achievement of autonomous driving systems in closed environments for unmanned ground vehicles (UGV), such as cars, ground robots, etc., is the LiDAR localization technology based on the prior map [1,2]. When the UGV system is in startup or tracking is lost, estimating the initial pose in the prior map is necessary [3]. Excellent initial pose estimation is a prerequisite for UGV to perform autonomous tasks and a guarantee for their safe operation. Initial pose estimation is typically accomplished using place recognition and point cloud registration techniques. Among them, place recognition utilizes the similarity between the current and map environments to archive. Therefore, the commonly used place recognition is processed by matching the global descriptor of the current LiDAR scan to the descriptor database of an a priori map, which was established previously, to find the optimal match with the maximum similarity score.

Existing LiDAR-based place recognition techniques only match the descriptor from a single keyframe with a prior map [4,5,6]. However, this method encounters several issues, such as limited feature information and susceptibility to error matching in similar environments, resulting in low place recognition accuracy, a high failure rate in initial pose estimation, and low system robustness. In some practical applications, researchers attempt to improve the accuracy by multiplying single-frame descriptor matches using consecutive keyframes during operation and selecting the result with the highest similarity score for place recognition. Nevertheless, this approach essentially involves simply repeating single-frame descriptor matching, offering a limited accuracy improvement.

This paper proposes a multi-frame descriptor-matching place recognition method based on the hidden Markov model (HMM) to address the above challenge. This method extends single-frame descriptor-matching place recognition to a technique that involves matching descriptors across multiple frames. During vehicle movement, the global descriptor information and pose transformation information from multiple consecutive keyframes online are used to model the HMM. We denote the pose as the state and node of HMM. The global descriptor-matching results will define the selection of hidden states and their probabilities. The pose transformations define the transition probability matrix for hidden states based on the spatial consistency [7]. The emission probabilities of the system are defined based on the established model, and the optimal matching is found by maximizing the emission probabilities to complete place recognition. This method effectively leverages more real-time keyframe information, significantly enhancing the place recognition accuracy and mitigating potential error-matching issues.

- We propose a place recognition method based on multiple descriptor matching, which utilizes the HMM model to integrate constraints from the SC descriptor and LiDAR odometry obtained from consecutive LiDAR keyframes online, aiming to maximize the emission probability for place recognition. Our approach significantly enhances the place recognition accuracy and reduces potential issues related to error matching compared to a single descriptor match.
- We validate our system based on the open-source KITTI dataset, and the results show that our method effectively improves the accuracy of place recognition.

## 2. Related Work

### 2.1. Initial Localization

Initial localization refers to estimating a robot or vehicle’s starting position and orientation within its environment. Initial estimation is crucial for the robot to begin mapping and navigating its surroundings accurately. Chen and Vizzo [8] proposed a Monte Carlo localization framework and utilized the Monte Carlo method to match the current LiDAR scan to a pre-built map for initial localization. Wasisto, Isro, and Istiqomah [9] utilized the adaptive Monte Carlo localization method for initial localization. However, the Monte Carlo method is computationally intensive; the real-time performance could be better. Many methods [4,10,11,12] are implemented or combine the initial localization through place recognition-based rough and point cloud-based fine matching. Our previous studies [10] utilized the intensity scan context algorithm for place recognition and the normal distributions transform (NDT) algorithm for point cloud registration. However, these methods only match the descriptor from a single keyframe with the prior map; this paper expands it to multiple descriptor matches for place recognition, which improves the place recognition accuracy. In addition, we use the NDT algorithm for point cloud registration.

### 2.2. Place Recognition

Place recognition methods are used to calculate the similarity between the current and historical environments. Numerous global place recognition techniques have been developed for mobile robots. Vision-based approaches typically employ the Bag-of-Words (BoW) model [13], which assesses the likeness between the current scene and the global map based on a pre-trained visual vocabulary [14,15]. Nevertheless, visual sensors are highly susceptible to variations in illumination and viewpoint. Despite efforts to mitigate this issue, visual-based methods often lack robustness in various scenarios. LiDAR-based approaches have garnered significant interest due to LiDAR’s resilience to variations in lighting and viewpoint. LiDAR-based place recognition methods utilizing LiDAR can roughly be categorized into three types: feature-based, global descriptor-based, and learning-based methods.

Feature-based methods involve extracting and matching point cloud features, similarly to the BoW method. Steder and Grisetti [16] matched a provided 3D scan with a database by leveraging point features, which were extracted through an improved Laplacian of Gaussian (LoG) method [17], and they evaluated the potential transformations based on the comparison of salient points within the scans. Zhuang and Cao [18] employed an image model called the bearing angle (BA) to transform LiDAR scans into 2D images. They then extracted ORB features and utilized a BoW approach for place recognition. Guo and Borges [19] proposed coupling the conventional geometric information from the LiDAR with its calibrated intensity return to achieve feature-based place recognition and proposed an ISHOT method to extract features, which is an improvement of SHOT [20]. However, the extraction of features usually requires extensive calculation.

Learning-based methods are drawing more attention at present. Uy et al. [21] extracted point cloud descriptors using deep learning, which combined the networks of PointNet [22] and NetVLAD [23]. Chen et al. [24] proposed OverlapNet, which uses different types of information, such as the intensity, normality, and semantics generated from LiDAR scans, to provide overlaps and relative yaw angle estimates between paired 3D scans. Zhou and Zhao [25] proposed an NDT-Transformer that learns a global descriptor from a set of 3D NDT cell representations. Luo et al. [26] utilized the NetVLAD network based on a bird’s eye view to achieve place recognition. They also proposed a rotation-invariant network called BEVPlace. Komorowski et al. [27] proposed a descriptor, named MinkLoc3D, to compute a discriminative 3D point cloud descriptor based on a sparse voxelized point cloud representation and sparse 3D convolutions. A shortcoming of these methods is that they require massive amounts of data and a long training period.

Compared to the above two methods, global descriptor matching is more efficient. Global descriptors utilize the geometric information of the environment for place recognition, which usually involves projecting 3D data into a 2D image matrix. Muhammad and Lacroix [28] introduced Z-projection, which combines 2D slice images along the z-axis to create a 2D image. Maximum intensity projection (MIP) selects each pixel’s highest gray level pixel value in the z-direction. M2DP [29] enhances this by considering information along the x- and y-directions perpendicular to the z-axis, improving the accuracy and reducing information loss and artifacts. The scan context (SC) descriptor [4] and its advancements [30] are considered state-of-the-art global descriptors. They convert LiDAR data into a 2D scan image, extract local features for representation, and employ a circular sequence for similarity matching.

SC employs a two-stage search algorithm for place recognition. Initially, it retrieves candidates using ring keys generated by SC, ensuring invariance to LiDAR viewpoint changes. Subsequently, the similarity score between the current scan and candidates is measured based on the SC distance. Finally, the optimal place index is selected among the candidates based on the similarity score. Much work has been conducted based on SC, aiming to replace the ring key to enhance its rotation-invariant retrieval abilities. For example, the original developer of SC [5] utilized a LeNet [31]-like network to retrieve the place index. Xu and Yin [32] transformed the features into the frequency domain and utilized the magnitude of the spectrum as the place descriptor, which was theoretically rotation-invariant. They both utilized SC to calculate the distance for the similarity score. Our method is applied for place recognition based on the SC method’s two-step search approach. Thus, it is compatible with SC and its extended variants. As a result, this work utilizes an SC descriptor as the global descriptor for discussion and experimental analysis.

## 3. System Overview

### 3.1. Definition and Notation

The mathematical symbols used in this paper are defined in the abbreviation table.

We define the skew-symmetric matrix of a vector $\omega \in R^{3}$ using ${\left(\cdot \right)}^{\wedge }$, as defined in Equation (1). Similarly, we can map a skew-symmetric matrix to a vector in $R^{3}$ using the operator ${\left(\cdot \right)}^{\vee }$.

(1) $$\begin{array}{c} \omega^{\wedge }={\left[\begin{array}{c} \omega_{1}\\ \omega_{2}\\ \omega_{3} \end{array}\right]}^{\wedge }=\left[\begin{array}{ccc} 0 & -\omega_{3} & \omega_{2}\\ \omega_{3} & 0 & -\omega_{1}\\ -\omega_{2} & \omega_{1} & 0 \end{array}\right] \end{array}$$

where ω, $\omega_{1}$, $\omega_{2}$, $\omega_{3}$ are used for indicators. Moreover, we expand ${\left(\cdot \right)}^{\wedge }$ and ${\left(\cdot \right)}^{\vee }$ to define the mapping between a special Euclidean group and its Lie algebra:

(2) $$\begin{array}{c} \xi^{\wedge }={\left[\begin{array}{c} \rho \\ \phi \end{array}\right]}^{\wedge }=\left[\begin{array}{cc} \phi^{\wedge } & \rho \\ 0^{⊤} & 0 \end{array}\right]\in R^{4\times 4} \end{array}$$

where ρ and ϕ are the position and posture components of ξ. Thus, the the exponential mapping between SO(3) and so(3), SE(3) and se(3) can be described as

(3) $$\begin{array}{c} \left\{\begin{array}{c} R=\exp(\phi^{\wedge })\\ \phi =\ln{\left(R\right)}^{\vee }\\ T=\exp(\xi^{\wedge })\\ \xi =\ln{\left(T\right)}^{\vee } \end{array}\right. \end{array}$$

### 3.2. System Structure

This work proposes an initial localization method. We match multiple global descriptors built from consecutive keyframes with a prior map for place recognition during the operation of the LiDAR SLAM system and then obtain the pose-by-point cloud registration. We follow the scan context (SC) [4] as a global descriptor in this work. Given that this study exclusively focuses on LiDAR keyframe poses, without addressing the velocity or IMU bias in real-time SLAM methods, we define the state as represented in Equation (4), where *p* represents the position and ϕ signifies the posture.

(4) $$x=\left[\begin{array}{c} p\\ \phi \end{array}\right]$$

**Figure 1:**
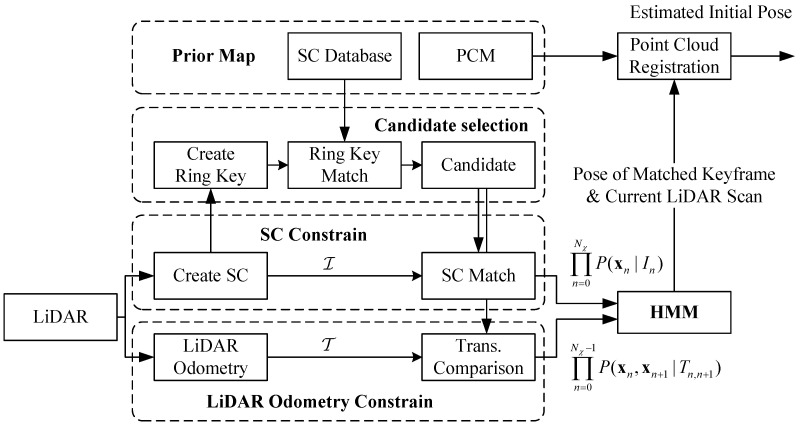
The structure of the system. I and T denote the observation of the SC and LiDAR odometry and are defined in Equation (10). $P(x_{n}|I_{n})$ and $P(x_{n},x_{n+1}|{\hat{T}}_{n,n+1})$ indicate the state estimation by the SC descriptor and LiDAR odometry and are defined in Equation (11). The system mainly comprises five components. Firstly, the candidate selection model searches for candidates for each online keyframe in the prior map using ring key descriptors, which are rotation-invariant. Next, the SC constraint model calculates the SC similarity scores of the candidates, while the LiDAR odometry constraint model computes the LiDAR odometry between the candidates of adjacent keyframes. The HMM model combines the SC and LiDAR odometry constraints for online states to achieve place recognition. Finally, we utilize the HMM results as an initial guess for point cloud registration and obtain the initial pose.

The structure of the proposed framework is shown in Figure 1; it mainly consists of five parts: the prior map, the candidate selection, the LiDAR odometry constraint, the SC constraint and the hidden Markov model (HMM).

The prior mapwas built previously and includes the SC database and point cloud map (PCM). While the robot is engaged in online movement, we utilize the multiple consecutive keyframes to build an SC descriptor and estimate the transformation. Then, we match the online SC to the SC database to search the candidates via candidate selection and calculate the similarity score via the SC constraint. We compare the online transformation to the prior map via the LiDAR odometry constraint. Moreover, we build the HMM model by combining the candidate selection and the SC constraint and achieve place recognition. Finally, we use the pose of the matched keyframe in the prior map, which is the place recognition result of the HMM, as an initial guess for point cloud registration and obtain the initial pose.

## 4. Prior Map

We established a prior map during the previous mapping process by following the keyframe-based LiDAR SLAM method [11,12,12]. For calculation efficiency, the prior map only preserves the data of all LiDAR keyframes. We adopt a simple but widely used approach in which we register a LiDAR frame as a keyframe when its pose exceeds a user-defined threshold compared to the previous keyframe. A lower threshold results in the selection of more keyframes and the more frequent sampling of the environment. This enhances the position recognition accuracy but also increases the search time during the recognition process. We set the threshold as 1 m, following the method of LIO-SAM [12].

### 4.1. PCM

The PCM is represented as a set of LiDAR keyframe scans, denoted as $F_{n}$, transformed into the corresponding map frame using their pose transformations in the map frame. Hence, we denote the state set and transformation set of these keyframes as M:

(5) $$\begin{array}{cc} M & =\left\{x_{n}^{M}|n=0,1,2,...,N_{M}-1\right\}\\ T_{M} & =\left\{T_{n}^{M}|n=0,1,2,...,N_{M}-1\right\} \end{array}$$

where $x_{n}^{M}$ denotes the state of the keyframe in the prior map, $T_{n}^{M}$ denotes the pose transformation of the keyframe scans in the map frame, and $N_{M}$ denotes the keyframe number. $x_{n}^{M}$ and $T_{n}^{M}$ can be obtained from the mapping process.

### 4.2. SC Descriptor Database

Subsequently, we construct SC descriptors for all keyframes, assembling the descriptor database for rough scan matches in online place recognition. Our creation of the SC descriptor follows the methodology outlined in the original paper [4], which we briefly introduce below.

**Figure 2:**
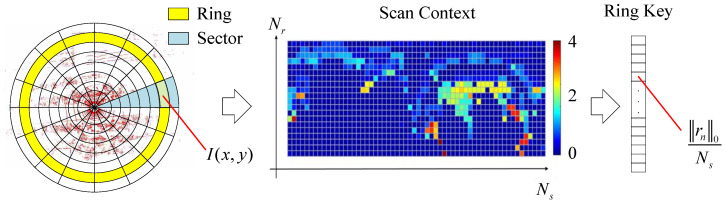
The schematic diagram of the SC descriptor [4]. The figure on the left illustrates the sampling of LiDAR scans using rings and sectors from a bird’s eye view, with the red color representing the point cloud of the LiDAR scan.. The middle figure illustrates the SC matrix. The figure on the right illustrates the ring key descriptor created from the SC.

Figure 2 shows the schematic diagram of the SC. The main idea of the SC is to project the 3D scan into a 2D coordinate from a bird’s eye view and divide it into azimuthal and radial bins in the sensor coordinate. Thus, the SC can be regarded as an egocentric place descriptor. The center of a scan acts as a global key point. Assume that $N_{s}$ and $N_{r}$ are the number of sectors and rings. We can easily create the feature image, as well as the SC description, represented as a $N_{r}\times N_{s}$ matrix *I*. The feature value of each bin is the maximum height of its points. Assuming that the point set of the bin in the *x* ring and *y* sector is $P_{x,y}$, the feature value is

(6) $$\begin{array}{c} I\left(x,y\right)=\left\{\begin{array}{c} \max_{p\in P_{x,y}}\;z\left(p\right),\;if\;P_{x,y}\neq \emptyset \\ 0,\;if\;P_{x,y}=\emptyset \end{array}\right. \end{array}$$

where z(·) is a function that returns the z-coordinate value of a point *p*. Furthermore, through the feature image *I*, the rotation-invariant sub-descriptor ring key vector *k* can be created as

(7) $$\begin{array}{c} k=RingKey\left(I\right)=\left(\frac{{∥r_{1}∥}_{0}}{N_{s}},\frac{{∥r_{2}∥}_{0}}{N_{s}},...,\frac{{∥r_{N_{r}}∥}_{0}}{N_{s}}\right) \end{array}$$

where $r_{i}$ denotes the row vector of the SC, and ${∥*∥}_{0}$ denotes the $L_{0}$ norm. The ring key vector can be seen as part of the SC. In this way, all keyframes can form the SC database set $I_{M}$, which will be used for online place recognition. It can be denoted as

(8) $$\begin{array}{cc} I_{M} & =\left\{I_{n}^{M}|n=0,1,2,...,N_{M}-1\right\}\\ K_{M} & =\left\{k_{n}^{M}=RingKey\left(I_{n}^{M}\right)|I_{n}^{M}\in I_{M}\right\} \end{array}$$

where $I_{n}^{M}\in R^{N_{r}\times N_{s}}$ and $k_{n}^{M}\in R^{N_{r}}$.

## 5. Online Place Recognition-Based HMM

### 5.1. HMM Model

The hidden Markov model (HMM) is a statistical model used to describe a Markov process with unobservable states, and it is mainly used in dealing with three problems: evaluation, decoding, and training [33]. Among them, the decoding problem is to estimate the optimal sequence of hidden states under the observation and transition probability, and we use it to model the place recognition process in this work.

**Figure 3:**
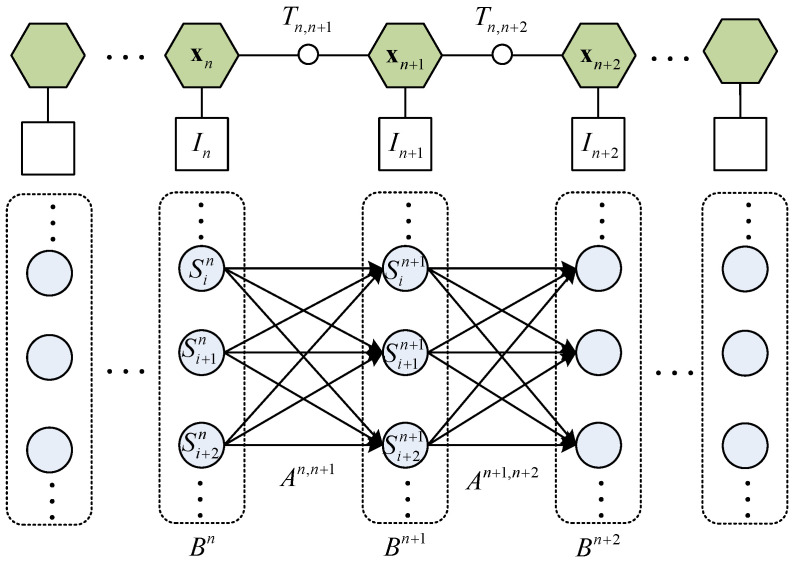
The structure of the HMM. x denotes the state of the online keyframe. *I* denotes the SC descriptor. *T* denotes the transformation between adjacent keyframes. *S* denotes the hidden state. *A* denotes the transition probability matrix, defined in Equation (22). The arrows between the hidden states illustrate the matrix, with the “length” of the arrow illustrating the spatial consistency between the actual transformation *T* and the transformation between hidden states. *B* denotes the hidden state probability vector, which is calculated through the similarity score of the SC match and is defined in Equation (16). x will be selected from the hidden states and minimize the path’s overall “length”.

The place recognition is processed by matching the scan context of the current LiDAR frame to the scan context database and finding the optimal match with the minimum distance. Our work extends the matching of individual LiDAR frames to multiple matches across consecutive LiDAR frames during motion, aiming to enhance the accuracy. This problem can be described as finding matches for those LiDAR frames in the descriptor database to maximize the emission probability of the whole process. The LiDAR frames to be matched can be seen as the nodes of the HMM, and the matches of each LiDAR frame can be seen as the hidden states of each node. We also compare the transformation between two nodes to the transformation between the hidden states of these two nodes. The transformation between two nodes can be obtained from the online LiDAR odometry, and the transformation of the hidden states can be obtained from the prior map. We take the comparison error as the constraint to reduce the false matches, and it can be normalized as the transition probability. As a result, the above process can be modeled as an HMM decoding problem and is illustrated in Figure 3.

While the robot is engaged in online movement, the state set of multiple consecutive keyframes to be matched can be modeled as an HMM and denoted as

(9) $$\begin{array}{c} \chi =\left\{x_{n}|n=0,1,2,...,N_{\chi }-1\right\} \end{array}$$

where $x_{n}$ denotes the state of the nodes in the HMM, and $N_{\chi }$ denotes the number of nodes. We build SC descriptors for all nodes through the method described in Section 4.2. The SC descriptor and LiDAR odometry are both obtained from online LiDAR scans and can be regarded as observations, and we mark them, respectively, as

(10) $$\begin{array}{cc} O & =\{I,T\}\\ I & =\left\{I_{n}|n=0,1,2,...,N_{\chi }-1\right\}\\ T & =\left\{{\hat{T}}_{n,n+1}|n=0,1,2,...,N_{\chi }-2\right\} \end{array}$$

where $I_{n}$ denotes the SC descriptor matrix of the nodes, and ${\hat{T}}_{n,n+1}$ denotes the transformation between the nodes. As a result, the problem of place recognition is to determine the nodes’ states in the prior map M, and the problem can be modeled as

(11) $$\begin{array}{cc} result & =\underset{\chi }{\arg\;\max}\;P\left(\chi |O\right)\\ \; & =\underset{\chi }{\arg\;\max}\;\prod_{n=0}^{N_{\chi }}P(x_{n}|I_{n})\prod_{n=0}^{N_{\chi }-1}P(x_{n},x_{n+1}|{\hat{T}}_{n,n+1}) \end{array}$$

where $P(x_{n}|I_{n})$ indicates the state estimation by the SC descriptor, and $P(x_{n},x_{n+1}|{\hat{T}}_{n,n+1})$ indicates the state estimation by online transformation.

### 5.2. Candidate Selection

The states in prior map M can be seen as the hidden states of each node. However, considering the computational efficiency, we only take a fixed number of hidden states as the candidates for each node, and they are selected via the ring key match [4].

Assume that the ring key descriptor of node $x_{n}$ is $k_{n}$:

(12) $$\begin{array}{cc} k_{n} & =RingKey(I_{n}) \end{array}$$

We match the $k_{n}$ with the descriptor database $K_{M}$ by the KD-tree to search the candidates with the highest similarity. The hidden state candidates of node $x_{n}$ can be marked as

(13) $$\begin{array}{c} S_{i}^{n},i=1,2,...,N_{cd} \end{array}$$

where $N_{cd}$ denotes the candidate number.

### 5.3. SC Constraint

The hidden state $S_{i}^{n}$ corresponds to one state in prior map M, assuming it as $x_{m}^{M}$. Then, we calculate the scan context distance *d* between the node $x_{n}$ and the candidates $S_{i}^{n}$. Their scan context descriptor can be written as $I_{n}$ and $I_{m}^{M}$. Because the scan context is very sensitive to rotation, we calculate the distance between $I_{n}$ and $I_{m}^{M}$ with all column shifts to find the best alignment, while determining the column shift is the same task as roughly aligning the point cloud of the LiDAR scan for yaw rotation at a $\frac{2\pi }{N_{s}}$ resolution; the minimum value can be seen as the final distance.

(14) $$\begin{array}{cc} \; & d=D\left(I_{n},I_{m}^{M}\right)=\min_{h\in [N_{s}]}\;d\left(I_{n},Shift\left(I_{m}^{M},h\right)\right)\\ \; & h^{*}=\underset{h\in [N_{s}]}{\arg\;\max}\;d\left(I_{n},Shift\left(I_{m}^{M},h\right)\right) \end{array}$$

where Shift(I,h) is defined as the *I* shifted for the *h* column clockwise. $h^{*}$ is denoted as the best column shift, and it can be used as in Section 5.4 to calculate the estimated pose of the hidden states. *d* indicates the similarity score between the scan context and the two places. The relationship between *d* and the similarity is the opposite; as a result, we use a natural exponential function to normalize *d* as the probability

(15) $$\begin{array}{c} P_{i}^{n}=P\left(x_{n}=S_{i}^{n}|I_{n}\right)=\frac{1}{e^{\lambda \cdot d}} \end{array}$$

where the parameter λ will affect the weight between the SC and odometry constraint, and it can be selected by practical application. The λ is generally within 1∼10, and we set it as 5 in this work. Through Equations (14) and (15), we can calculate the probabilities for all hidden states and form the hidden state probability vector for node $x_{n}$ as

(16) $$\begin{array}{c} B^{n}=\left[P_{1}^{n},P_{2}^{n},...,P_{N_{cd}}^{n}\right] \end{array}$$

### 5.4. LiDAR Odometry Constraint

Assume that the transformation between two continuous nodes $\left[x_{n},x_{n+1}\right]$ is $T_{n,n+1}$, and its Lie algebra is $\xi_{n,n+1}$. The observation of $T_{n,n+1}$ is ${\hat{T}}_{n,n+1}$, denoted in Equation (10), and we denote the corresponding Lie algebra as ${\hat{\xi }}_{n,n+1}$, so we have

(17) $$\begin{array}{c} {\hat{\xi }}_{n,n+1}=\xi_{n,n+1}+\delta {\hat{\xi }}_{n,n+1} \end{array}$$

where $\delta {\hat{\xi }}_{n,n+1}$ is the noise; it obeys the zero mean multivariate Gaussian distribution and can be described by the covariance matrix $Q\in R^{6\times 6}$. Under the observation ${\hat{T}}_{n,n+1}$, the posterior probability density of $T_{n,n+1}$ can be written as

(18) $$\begin{array}{c} P\left(\xi_{n,n+1}|{\hat{\xi }}_{n,n+1}\right)=N\left(\xi_{n,n+1};{\hat{\xi }}_{n,n+1},Q\right)=\frac{1}{\sqrt{\left|2\pi Q\right|}}\exp\left\{-\frac{1}{2}{∥\xi_{n,n+1}-{\hat{\xi }}_{n,n+1}∥}_{Q}^{2}\right\} \end{array}$$

We utilize Equation (18) to analyze the transformation between the hidden states of two nodes, assuming that they are $S_{i}^{n}$ and $S_{j}^{n+1}$. From Section 4, we know that the corresponding pose can be obtained from the prior map, denoted as $T_{i}^{M}=\left[R_{i}|p_{i}\right]$ and $T_{j}^{M}=\left[R_{j}|p_{j}\right]$. Combined with the column shift from Equation (14) as $h_{i}^{*}$ and $h_{j}^{*}$, the estimated transformation can be written as

(19) $$\begin{array}{cc} {\tilde{T}}_{i} & =[Rot\_z\left(h_{i}^{*}\cdot \frac{2\pi }{N_{s}}\right)R_{i}|p_{i}]\\ {\tilde{T}}_{j} & =[Rot\_z\left(h_{j}^{*}\cdot \frac{2\pi }{N_{s}}\right)R_{i}|p_{j}]\\ Rot\_z(\psi ) & =\left[\begin{array}{ccc} \cos\psi & -\sin\psi & 0\\ \sin\psi & \cos\psi & 0\\ 0 & 0 & 1 \end{array}\right] \end{array}$$

where Rot_z(⋅)∈SO(3) denotes the rotation matrix around the z-axis, and the input variable is the rotation angle in radians. ψ is used for the indicator. Thus, the transformation between them can be written as

(20) $$\begin{array}{cc} {\tilde{T}}_{ij} & ={\tilde{T}}_{i}^{-1}{\tilde{T}}_{j}\\ {\tilde{\xi }}_{i,j} & =ln{\left({\tilde{T}}_{ij}\right)}^{\vee } \end{array}$$

Then, we substitute ${\tilde{\xi }}_{i,j}$ into Equation (18) to calculate the probability:

(21) $$\begin{array}{c} P_{i,j}^{n,n+1}=P\left({\tilde{\xi }}_{i,j}|{\hat{\xi }}_{n,n+1}\right) \end{array}$$

This can be seen as the transition probability between the two hidden states. Through the above process, we can obtain the transition probabilities between all candidates of the two nodes and form the transition probability matrix:

(22) $$\begin{array}{c} A^{n,n+1}=\left[\begin{array}{cccc} P_{0,0}^{n,n+1} & P_{1,0}^{n,n+1} & ... & P_{N_{cd},0}^{n,n+1}\\ P_{0,1}^{n,n+1} & P_{1,1}^{n,n+1} & ... & P_{N_{cd},1}^{n,n+1}\\ ... & ... & ... & ...\\ P_{0,N_{cd}}^{n,n+1} & P_{1,N_{cd}}^{n,n+1} & ... & P_{N_{cd},N_{cd}}^{n,n+1} \end{array}\right] \end{array}$$

### 5.5. Place Recognition and Point Cloud Registration

After obtaining the hidden state probability and the transition probability between the hidden states of all nodes, combining Equation (11), we can obtain the emission probability of the HMM. We find an optimal path to maximize the overall emission probability, and the node states can indicate the recognized place. Reflected on the image, as shown in Figure 3, it represents the overall “length” of the selected hidden state line from $x_{0}$ to $x_{N_{\chi }-1}$. Due to the small numerical value of the number of states, we use an exhaustive method to find the optimal path of the HMM (with the highest overall probability). At this point, the hidden states of each node are the matching results.

After obtaining the matching results, we obtain the rough pose of the online keyframe. We use this pose as the initial guess for point cloud registration. Based on the point cloud registration algorithm, we match the LiDAR point cloud of the online keyframe with the prior map PCM to obtain an accurate 6-degree-of-freedom pose estimation. In this work, we utilize the normal distributions transform algorithm [34] for point cloud registration.

The overall procedure is represented in pseudocode in Algorithm 1.

**Algorithm 1** Online Place Recognition Procedure**Input** online LiDAR keyframe scans $F_{n}$, SC and ring key descriptor of prior map $I_{M}$ and $K_{M}$ **Output** Initial Pose
1:KFNum = 02:**while** 1 **do**3:  **if** Received keyframe scans $F_{n}$ **then**4:    KFNum++5:    Build SC and ring key descriptor for $F_{n}$ by Equations (6) and (7)6:    Select candidates by ring key-based global place retrieval7:    Calculate the probability for all hidden states by Equation (15).8:    Form the hidden state probability vector $B^{n}$.9:    **if** KFNum>=2 **then**10:      Calculate the transformation between $F_{n-1}$ and $F_{n}$ through F-LOAM [11]11:      Calculate the transition probability matrix $A^{n,n+1}$12:    **end if**13:    **if** KFNum>=$N_{\chi }$ **then**14:      Place recognition through HMM.15:      Obtain initial pose through point cloud registration.16:      **return** initial pose.17:    **end if**18:  **end if**19:**end while**


## 6. Experiments

### 6.1. Experiment Preparation

To validate our approach, we performed experiments using the KITTI dataset (https://www.cvlibs.net/datasets/kitti/, accessed on 10 June 2022), which is public and widely acknowledged as a benchmark dataset for the evaluation of autonomous driving systems. It comprises 22 sequences, with over 43,000 scans captured using a Velodyne HDL-64E LiDAR launched by Velodyne Lidar, Inc. (San Jose, CA, USA). This dataset encompasses various outdoor scenarios, such as urban roads, country roads, and highways. Sequences 0–10 also provide GNSS-INS ground truth pose information. We specifically utilized sequences 05, 06, and 08 from the KITTI Odometry dataset for our experiments. These sequences feature paths with a significant number of overlapping areas, making them suitable for path recognition experiments. To perform the place recognition experiments, we divided the dataset into two distinct parts, target and source, and we matched the keyframes in the source part with those in the target one. In Table 1, we provide details regarding the period, length, and number of keyframes for each part; we also provide the period in the original dataset in parentheses.

**Figure 4:**
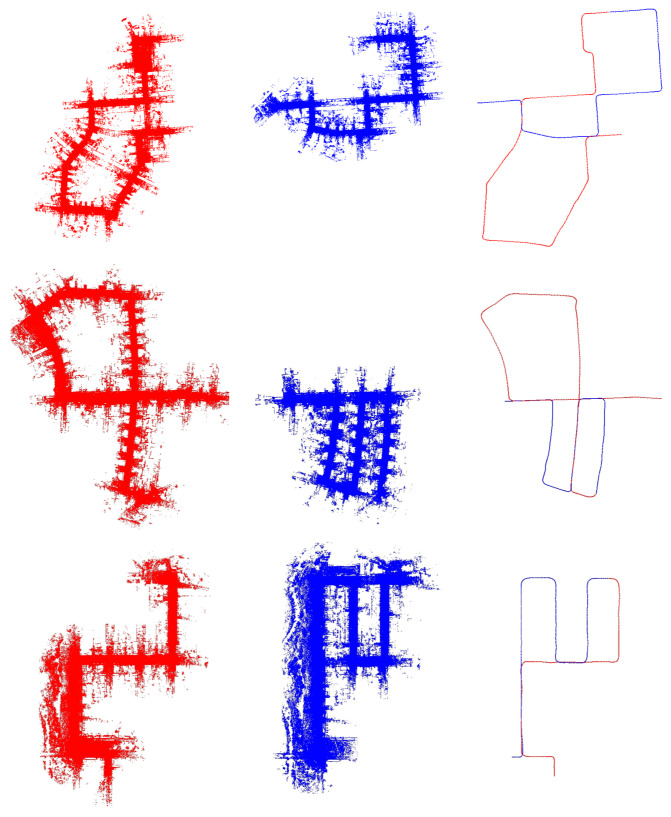
The dataset illustration. The first, second, and third lines represent the 00, 05, and 08 sequences of KITTI, respectively. The red represents the target, while the blue represents the source. The figures on the left show the target PCM, while the middle figures show the source PCM. The figures on the right show the paths of both.

We used the state-of-the-art feature-based LiDAR odometry method proposed in F-LOAM [11] to provide the LiDAR odometry for the target and source data. To display the dataset’s overlapping areas, we show the point cloud map and the target and source pose in Figure 4.

**Figure 5:**
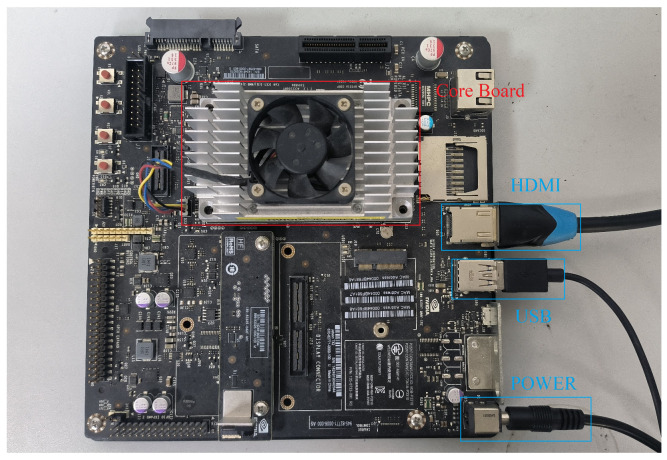
Physical image of TX2. The TX2 consists of a core board and a peripheral board. The core board houses the chips, memory, and storage. The peripheral board can be customized according to the functionality, primarily comprising a power supply and interface extensions.

The proposed method is implemented in C++ and integrated into Ubuntu 16.04 and the robot operating system (ROS) on an NVIDIA Jetson TX2. The Jetson TX2 is an embedded artificial intelligence computing platform launched by NVIDIA (Santa Clara, CA, USA); it comes with a built-in GPU and CPU, with 8 GB of memory by Samsung Electronics (Suwon, Republic of Korea) and 32 GB of storage capacity by SanDisk (Milpitas, CA, USA). The TX2 contains a dual-core NVIDIA Denver 2 64-bit CPU and quad-core Arm (Cambridge, UK) Cortex-A57 MPCore processor, the operating frequency of the Denver 2 is 2 GHz and that of the Cortex-A57 is 2 GHz. A physical image of the TX2 is shown in Figure 5.

### 6.2. Precision Recall Evaluation

First, we establish SC descriptors for the target and source keyframes separately. The number of sectors and rings will affect the resolution of the SC descriptor, influencing the balance of the computational efficiency and the quality of the SC descriptors. We set the number of sectors and rings for the SC descriptor as 20 and 60, which follow the original paper [4]. The sampling range will affect the quality of the SC descriptor. Due to occlusion, distant point clouds are sparse. When the sampling distance is too large, it can cause many bins to have no point clouds, decreasing the quality of the SC descriptors. However, when the sampling distance is too small, the utilization of environmental information becomes smaller, also resulting in a decrease in the quality of the SC descriptors. We set the sampling range to 80 m according to the dataset’s sampling environment.

Then, we match the source descriptor with the target descriptor individually to find the optimal match. As this work is an extension of the SC, the SC has already been compared to algorithms such as M2DP, Z-projection, and ESF in [4]. Therefore, this work only conducts a comparative analysis with the SC. We denote the proposed method in this work as HMM. We also conduct a comparative analysis with Mul SC, which utilizes the simple repetition of single-frame descriptor matching for the nodes in the HMM and chooses the one with the best similarity score. Thus, Mul SC can be used in an ablation experiment for the HMM.

The hidden states are the candidates for the SC match, which is selected by the ring key. Thus, the hidden state number $N_{cd}$ will affect the balance of the computational efficiency and the accuracy of place recognition in the SC matching process. We set the hidden state number $N_{cd}$ as 5 for all methods according to the experimental situation. The selection of the node number $N_{\chi }$ and the distance between nodes will impact the performance of the HMM. Specifically, $N_{\chi }$ will affect how much environmental information we use in our methods. When $N_{\chi }$ is larger, we will use more environmental information, and the precision will improve; however, it requires a long operation distance for place recognition, and each node must be correctly identified or the recall will be reduced. The distance between the nodes also affects the accuracy of place recognition. When the distance between the nodes is relatively small and less than the sampling distance, adjacent nodes have overlapping areas in their environmental sampling. Consequently, as the distance between the nodes increases, the environmental difference between the two nodes becomes greater, and more environmental information is utilized by the HMM. However, if the distance becomes too large, the overlapping areas diminish, causing the environmental information used to increase slowly or not at all, while the required operation distance for place recognition increases. In this work, we set the node number $N_{\chi }$ for the HMM and Mul SC as 3 and the distance between the nodes as 5 m according to the experimental situation.

We use the precision–recall curve and the corresponding area under the curve (AUC) to evaluate place recognition. The precision and recall are defined as

(23) $$\begin{array}{cc} \; & Precision=TP/(TP+FP)\\ \; & Recall=TP/(TP+FN) \end{array}$$

where TP, FP, and FN denote the number of place recognition results that are true positives, false positives, and false negatives, respectively. The prediction results’ classification is shown in Table 2.

**Figure 6:**
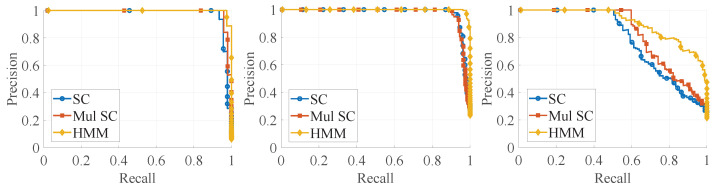
The precision–recall curve. The left, middle, and right images represent the 00, 05, and 08 sequences of KITTI, respectively. The horizontal axis represents the recall rate, while the vertical axis represents the precision rate. We have plotted the precision–recall curves for the SC, Mul SC, and HMM methods. The precision–recall curves for the HMM method consistently lie above those of the other methods, and, in almost all cases, they completely encompass the other curves.

The detection is considered a true positive if the ground truth distance between the query and the matched node is less than 5 m. The precision–recall curve is shown in Figure 6. The AUC is shown in Table 3.

Accuracy represents the probability of correctly predicting a loop, while recall represents the probability that the predicted loop is correct. In an ideal environment, both the accuracy and recall can reach 1 simultaneously, but, in reality, it is difficult for both to reach 1 simultaneously. Therefore, a higher precision–recall curve indicates a better method. Additionally, a larger area under the curve (AUC) signifies better performance. From Figure 6, it can be seen that the precision–recall curves of the HMM are all above those of the other methods, and almost all of them can completely contain the other curves. Moreover, Table 3 shows that the AUC of the HMM is the largest for each sequence. Therefore, our method can improve the performance of place recognition compared to the original SC method, with an average performance improvement of 5.8% and with a maximum improvement of 15.3%. The experimental results show that the method proposed in this paper can not only achieve place recognition, but the vast majority of the results are also correct. This is of great significance for the initial localization.

### 6.3. Time Consumption

The time consumption is shown in Table 4. We provide the values of the maximum (Max), mean (Mean), and standard deviation (Std). The Max and Mean values show that the time consumption of the HMM and Mul SC is the same, and both values are $N_{\chi }$ times the SC. Due to the fact that the ring key search and SC match time of the Mul SC and HMM methods are also consumed during their respective keyframe cycles, which is the same as in the SC method, the increased computational complexity of the HMM method mainly lies in the HMM recognition. From the comparison of the time consumption of Mul SC and the HMM, it can be seen that the time is mainly spent on the ring key search and SC matching, while HMM recognition requires very little computation, accounting for approximately 0.6% of the total time. Therefore, compared to the original SC method, the additional computational cost of the HMM method is very small.

### 6.4. Running Distance

**Figure 7:**
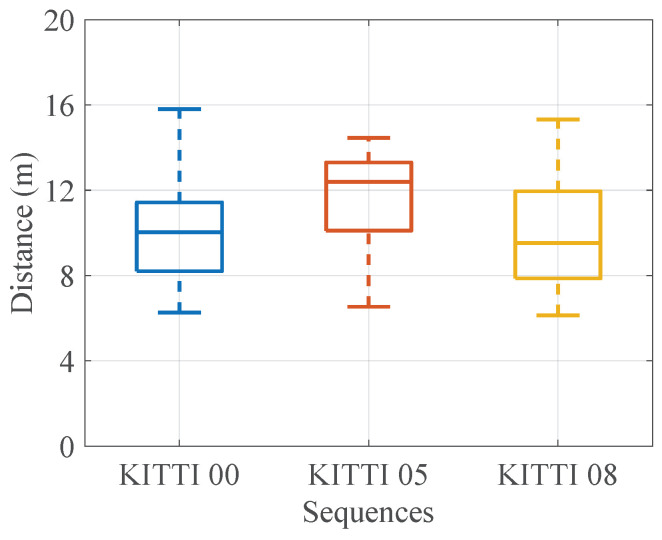
HMM running distance. We use a box chart to show the distribution of the running distance, and it can be seen that the average running distance is about 10 m, with a maximum of no more than 16 m.

Due to the fact that the method proposed in this work requires the system to run for a certain distance to estimate the initial pose, similar to some Monte Carlo-based place recognition methods [8,9], this paper evaluates the required distance. Figure 7 shows the HMM running distance; it can be seen that the maximum distance is less than 16 m. According to actual engineering experience, the convergence distance of the 3D Monte Carlo localization method is usually around 10–50 m. Thus, the proposed method only requires a short running distance to achieve good results.

## 7. Conclusions

In this paper, we utilize the HMM algorithm to expand single SC matching to multiple SC matching for place recognition while a robot is moving. Compared to the original method, our method showed higher place recognition performance across the dataset sequences, with an average performance improvement of 5.8% and with a maximum improvement of 15.3%. We improved the SC matching performance under an acceptable running distance of 16 m, and it only increased the time consumption by approximately 0.6%. Therefore, our method improves the performance of existing methods.

Although our method improves the performance, the manual selection of the number of nodes and the distance between the nodes significantly impacts it, making it inefficient in practical operation. In future work, we plan to extend our method to adaptively select the number of nodes and the distance between nodes based on the environment. In this way, our method can become more efficient and widely used.

Our method can also be extended to LiDAR surveying scenes with non-moving objects, leveraging the HMM framework to detect and distinguish between static and dynamic elements within the scene, as demonstrated in the study by Chau et al. [35]. Further analysis of this application will be conducted in future work.

## Figures and Tables

**Table 1:** KITTI dataset details.

Sequence	Sub-Map	Time Period (s)	Length (m)	Keyframes
KITTI-00	Target	110 (0–110)	768.99	476
	Source	150 (110–260)	1123.99	679
KITTI-05	Target	125 (0–125)	970.91	538
	Source	162 (125–287)	1330.38	687
KITTI-08	Target	110 (0–110)	919.30	500
	Source	100 (110–210)	696.50	420

**Table 2:** Classification of prediction results.

Classification	Predict True	Predict False
Actual True	True Positive (TP)	False Negative (FN)
Actual False	False Positive (FP)	True Negative (TN)

**Table 3:** Area under the curve (AUC) (ratio).

Sequence	SC	Mul SC	HMM
KITTI 00	0.9549	0.9667	**0.9722**
KITTI 05	0.9707	0.9675	**0.9919**
KITTI 08	0.7754	0.8193	**0.8946**

Bold indicates the best result of all methods.

**Table 4:** Time consumption (ms).

Sequence	SC			Mul SC			HMM		
	Max	Mean	Std	Max	Mean	Std	Max	Mean	Std
KITTI 00	1.0132	0.8372	0.0250	2.8615	2.5116	0.0498	2.8789	2.5295	0.0500
KITTI 05	1.0016	0.8265	0.0200	2.7973	2.4782	0.0375	2.8164	2.4953	0.0376
KITTI 08	1.0894	0.9492	0.0249	3.1127	2.8477	0.0501	3.1322	2.8652	0.0500

## Data Availability

Data are contained within the article.

## Abbreviations

SO(3)	Special Orthogonal Group
SE(3)	Special Euclidean Group
so(3)	Lie Algebra of SO(3)
se(3)	Lie Algebra of SE(3)
$p\in R^{3}$	Position Vector
R∈SO(3)	Rotation Matrix
T∈SE(3)	Transformation Matrix
ϕ∈so(3)	Lie Algebra of *R*
ξ∈se(3)	Lie Algebra of *T*
